# Preexisting chronic pain is not associated with moderate-to-severe acute pain after laparoscopic cholecystectomy: a prospective cohort study

**DOI:** 10.1097/PR9.0000000000001214

**Published:** 2024-11-13

**Authors:** Bishal Nepali, Asish Subedi, Krishna Pokharel, Ashish Ghimire, Jagat Narayan Prasad

**Affiliations:** a Panchthar District Hospital, Phidim, Panchthar, Nepal; b BP Koirala Institute of Health Sciences, Dharan, Nepal

**Keywords:** Chronic pain, Laparoscopic cholecystectomy, Postoperative pain

## Abstract

**Introduction:**

We aimed to evaluate the relationship between preexisting chronic pain and acute postoperative pain after laparoscopic cholecystectomy (LC) and to identify predictors of acute postsurgery pain.

**Methods:**

In this prospective cohort study, patients undergoing LC with general anesthesia were enrolled. The primary outcome was the incidence of moderate-to-severe pain during movement in the first 24 hours after surgery. Logistic regression analysed the association of perioperative risk factors with acute postoperative pain. Model performance was assessed using receiver operating characteristic curves (ROC), calibration belt plots, and 10-fold cross-validation.

**Results:**

Of the 193 patients analysed, 49.74% experienced moderate–severe acute pain. Univariable analysis indicated that preoperative chronic pain increased the likelihood of acute postoperative pain (odds ratio [OR], 2.53; 95% confidence interval [CI], 1.40–4.55). Risk factors identified in the multivariable analysis were higher preoperative pain intensity (OR, 1.28; 95% CI, 1–1.65), sleep disturbances (OR, 1.42; 95% CI, 1.24–1.61), intraoperative fentanyl supplementation (OR, 3.68; 95% CI, 1.48–9.12), incision extension for gallbladder retrieval (OR, 7.27; 95% CI, 1.58–33.39), and abdominal drain placement (OR, 6.09; 95% CI, 1.08–34.34). Dexamethasone use was associated with reduced pain (OR, 0.05; 95% CI, 0.004–0.74). The model demonstrated strong discrimination (ROC = 0.909) and good calibration (test statistic = 0.02; *P* = 0.89). Cross-validation showed slightly lower accuracy (ROC = 0.896).

**Conclusions:**

Preexisting chronic pain was not associated with acute postoperative pain following laparoscopic cholecystectomy. Significant predictors for acute pain included higher preoperative pain intensity, sleep disturbances, intraoperative fentanyl, incision extension, and abdominal drain placement, while dexamethasone use was associated with reduced pain.

## Abstract 2

Supplemental Digital Content is Available in the Text.

Preoperative pain intensity, sleep disturbances, intraoperative fentanyl, incision extension for gallbladder retrieval, and abdominal drains predict acute pain after laparoscopic cholecystectomy, while dexamethasone reduces it.

## 1. Introduction

Although laparoscopic cholecystectomy (LC) is minimally invasive, early postoperative intense pain is the most common complaint after the procedure.^20,35^ Following LC, 26% to 60% of patients experience moderate-to-severe pain within the first 24 hours.^3,10,16,33^ As a result, a multimodal analgesic strategy is recommended to minimize pain after LC. Despite the use of perioperative multimodal analgesic treatment, acute pain continues to be a significant issue. Pain after LC is multifactorial, resulting from surgical trauma to the abdominal wall at the port sites, the local effect of carbon dioxide on the peritoneum, and the distention of the abdominal wall and diaphragm.^21^

Owing to inadequate pain control, hospital stays and the convalescence period after LC are prolonged.^3,4^ Moreover, poorly controlled postoperative pain has several unwanted effects.^5,8^ Therefore, if we could predict which patients will experience more postoperative pain, a more aggressive pain management strategy could be adopted perioperatively. This would not only improve patient satisfaction but also prevent poor outcomes, such as chronic pain after surgery.

Among several risk factors, preexisting pain has been shown to predict the intensity of acute postoperative pain after various surgeries.^32,37^ However, regarding preexisting pain before LC and its association with acute postoperative pain, only a few studies have examined this aspect with contradicting results.^3,35^ Thus, our primary objective was to test the hypothesis that preexisting preoperative chronic pain is a risk factor for dynamic moderate-to-severe acute pain during the first 24 hours after LC. The secondary objectives were to examine baseline demographic, clinical, and psychobehavioral features and intraoperative factors in relation to dynamic moderate-to-severe pain after LC. In addition, the aim of this study was to identify perioperative predictors of acute postsurgical pain following LC.

## 2. Methods

This prospective cohort study was conducted at BP Koirala Institute of Health Sciences (BPKIHS) between September 2022 and June 2023. After the study protocol was approved by the BPKIHS institutional review committee (IRC/2283/022), it was registered at ClinicalTrials.gov (principal investigator: Dr. Bishal Nepali, ClinicalTrials.gov ID NCT05543668) before patient enrollment. All patients provided written informed consent before beginning the study procedure. This study adhered to the STROBE (Strengthening the Reporting of Observational Studies in Epidemiology) guidelines.^38^

We recruited patients aged 18 years or older with the American Society of Anesthesiologists (ASA) physical status 1, 2, or 3 who were scheduled for LC. Exclusion criteria included patients with cognitive impairments (lack of capacity to provide informed consent), inability to understand the Nepali language, severe psychiatric or neurologic disorders, pregnancy, acute cholecystitis managed conservatively, choledocholithiasis, features of obstructive jaundice, and those whose LC was converted to open procedure. During the presurgical visits in the evening before surgery, all eligible patients were informed about the study and its procedures. The presurgical assessment and data collection were conducted by the principal investigator. Baseline patient information such as age, sex, body mass index (BMI), ethnicity, marital status, education, occupation, socioeconomic status (assessed according to Kuppuswamy's socioeconomic scale adjusted for Nepal),^12^ smoking status, alcohol intake, insurance coverage, Charlson comorbidity index, ASA physical status classification, and operative indication was recorded.

Preoperative use of pain medications (ie, acetaminophen, opioids, NSAIDs, gabapentinoids) was recorded. Before surgery, patients were instructed on how to use a Nepali version of the 10-cm numeric rating scale (NRS) for measuring postoperative pain,^29^ with the endpoints labeled as “no pain” and “the worst possible pain.” The following questionnaires were administered in a face-to-face interview by the principal investigator: presence of preexisting chronic pain at the site of surgery and/or another site for more than 3 months^34^; patient-reported outcomes measurement information system (PROMIS) pain intensity score^30^; patients' expectations of pain during the first 24 hours postoperatively on the NRS scale (mild, moderate, or severe pain); patients' levels of preoperative pain and its impact on four domains (PROMIS pain interference, PROMIS pain behavior, PROMIS depression, and PROMIS sleep disturbance), assessed using the Nepali version of the PROMIS short form.^30^ The assessment of psychological vulnerability included measuring anxiety using the Nepali version of the Amsterdam Preoperative Anxiety and Information Scale,^25^ pain catastrophizing using the Nepali version of the pain catastrophizing scale,^31^ neuroticism,^19^ and pain sensitivity, which was assessed using the NRS scale by applying a tourniquet with a pressure of 250 mm Hg on the patient's nondominant arm for 5 minutes.

The anesthesia, surgical procedure, and perioperative pain management were standardized. No premedication was used. In the operating room, standard monitors were applied. General anesthesia was induced with IV fentanyl 1.5 µg/kg and propofol 2 to 2.5 mg/kg until cessation of verbal response. Endotracheal intubation was facilitated with vecuronium 0.1 mg/kg or rocuronium 0.75 to 1 mg/kg. The lungs were mechanically ventilated using a circle system with a mixture of 50% oxygen and air to maintain end-tidal CO_2_ between 35 and 45 mm Hg. Intravenous paracetamol 1 g was infused over 15 minutes after induction of anesthesia. Patients received IV dexamethasone 8 mg. Preincisional infiltration at the port site was done with 20 mL (100 mg) of 0.5% plain bupivacaine (6 mL each for the epigastric and umbilical port, and 4 mL for each working port). Anesthesia was maintained with isoflurane or sevoflurane.

Neuromuscular blockade was maintained with supplemental doses of vecuronium or rocuronium after observing the curare notch in the capnograph. If required, supplemental fentanyl boluses were given at the discretion of the attending Anesthesiologist. Any deviations from the perioperative analgesic regimen were noted. Hasson's surgical technique was used. Pneumoperitoneum was achieved with CO_2_ while maintaining intraabdominal pressure below 15 mm Hg. All patients received IV ketorolac 30 mg and ondansetron 4 mg after removal of the gallbladder. Local lavage with saline followed by suction was performed after gallbladder removal. At the end of the surgery, any remaining CO_2_ in the peritoneal cavity was expelled by manual compression of the abdomen with open trocars. The operating surgeon assessed the intraoperative anatomical status and inflammation of the gallbladder based on the Parkland grading scale used for cholecystitis severity criteria.^17^ The extension of an incision, placement of closed suction abdominal drains, and conversion to open surgery were recorded.

The patients were asked to rate their postoperative pain (incisional and/or intraabdominal pain) at rest and during dynamic states (pain from coughing, moving, or deep inspiration was regarded as dynamic pain) in the postanesthesia care unit (PACU; 30 minutes, 1 hour) and in the inpatient surgical unit (2, 6, 12, and 24 hours) using the 10-cm NRS scale. Shoulder pain was also assessed at the same time points. Postoperative pain management included IV paracetamol 1 g every 6 hours and ketorolac 30 mg every 8 hours. In the PACU, if the NRS pain score was greater than 3, IV morphine 2 mg was given and repeated every 5 minutes until the NRS pain score was 3 or less. The patient was transferred to the surgical inpatient unit after a 1-hour stay in the PACU. In the surgical inpatient unit, 50 mg tramadol IV was administered as needed to maintain an NRS pain score of 3 or less in the first postoperative 24 hours (maximum tramadol dose 300 mg). Postoperative opioid consumption up to 24 hours after surgery was reported in oral morphine equivalents (https://globalrph.com/medcalcs/opioid-conversions-calc-original-single-agent/). The primary outcome measure was dynamic moderate-to-severe abdominal pain in the first postoperative 24 hours as measured by the NRS scale.

The incidence of postoperative moderate-to-severe pain after LC was estimated to be between 26% and 60%.^3,10,16,33^ Based on these studies, we estimated an approximate 40% occurrence of moderate-to-severe pain after LC. The sample size was calculated based on 10 events per risk variable in logistic regression.^24^ We used the formula N = (n × 10)/I, where N = the required sample size, n = number of variables to be tested, and I is the incidence of the primary outcome. The number of potential variables to be included in the model was 7 (age, sex, preexisting preoperative pain, intensity of preoperative pain, pain catastrophizing, preoperative pain sensitivity, and preoperative anxiety). With 10 events per covariate, this would require 70 events; 70 events divided by the odds of occurrence of moderate-to-severe postoperative pain resulted in 70/0.40 = 175. We expected a 10% dropout rate during the study. Based on the formula for adjusted sample size = calculated sample size/(1 − dropout rate), finally, we planned to recruit 195 participants.

Data normality was assessed by skewness and kurtosis, the Shapiro-Wilk test, and visually inspecting histograms. Patient data were presented as the mean (standard deviation), median (interquartile range [IQR]), or frequency (percentage). Student unpaired *t* test was used to compare continuous data with a normal distribution between the 2 groups (patients with and without preexisting chronic pain), while the Mann-Whitney test was applied for data that were not normally distributed. The Chi-square tests were applied for categorical variables, with Fisher's exact tests used when the expected cell counts were below 5. Univariable and multivariable logistic regression analyses were performed to explore the association between preoperative chronic pain and moderate-to-severe acute pain after LC. For building the multivariable logistic regression model, automated backward stepwise selection was used, and covariates with *P* < 0.1 were added to the model. The results were reported as odds ratios with 95% confidence intervals (CIs). The degree of multicollinearity was assessed using the variance inflation factor (VIF), and variables with VIF values > 5 were dropped. Model discrimination for dynamic moderate-to-severe pain was assessed using the area under the receiver-operator characteristic curve (ROC), with values > 0.80 reflecting excellent discrimination.^1^

We performed internal validation of the final model using 10-fold cross-validation to produce a mean area under the curve (AUC) as an indicator of prediction accuracy. Bootstrapped bias-corrected 95% confidence intervals for the AUC were also calculated. Calibration of the model was evaluated using the Hosmer–Lemeshow goodness-of-fit test. Furthermore, the relationship between predicted and observed risk of the outcome was evaluated using a calibration belt plot, which graphically shows whether the observed responses significantly differed from expected probabilities and depicts the direction of deviation (miscalibration) at 95% and 99% confidence levels.^22^ The presence or absence of any substantial deviations from the 45° line of perfect fit was assessed with a calibration test. A *P*-value less than or equal to 0.05 was considered statistically significant. We used Stata (Software for Statistics and Data Science) Version 15.0 (StataCorp LP, College Station, TX) for all analyses.

## 3. Results

**Figure 1.**
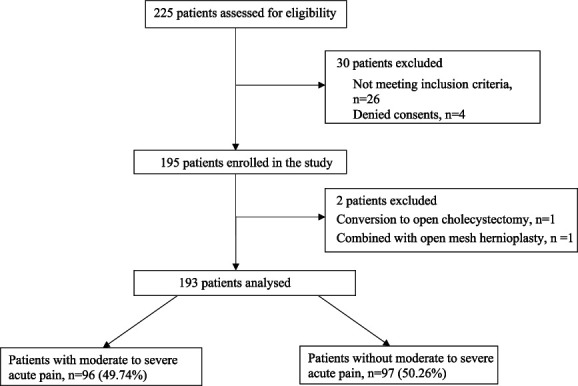
Flow diagram of the study.

A total of 225 patients planned for elective LC under general anesthesia were assessed for eligibility. Out of the 225 patients, 30 were excluded, with 26 patients not meeting the inclusion criteria and 4 patients declining consent. The remaining 195 patients were enrolled. Two patients were excluded from the analysis: one case was converted to open cholecystectomy, and another case was combined with open mesh hernioplasty. Thus, 193 patients were finally analyzed (Fig. 1).

Among the 193 participants, 112 (58%) had no preexisting chronic pain, and 81 (42%) were experiencing chronic pain (pain duration >3 months). Overall, 11 (5.70%) patients experienced moderate-to-severe acute postoperative pain at rest, whereas 96 (49.74%) patients reported moderate-to-severe pain during movement. Similarly, 17 (8.81%) patients reported shoulder pain after LC. Baseline characteristics, intraoperative, and postoperative profiles of study participants, stratified by the presence or absence of preoperative chronic pain, are depicted in Table 1. A weak correlation was found between preoperative pain intensity and sleep disturbances (*r* = 0.25).

**Table 1:** Demographic and perioperative characteristics of study participants, stratified by presence of preoperative chronic pain.

Variables	All patients (N = 193)	Preexisting chronic pain		
		No (N = 112)	Yes (N = 81)	*P*
Age (in y)	45.77 ± 14.84	43.76 ± 15.01	48.55 ± 14.23	0.026
Female	150 (78%)	86 (77%)	64 (79%)	0.714
Occupation				
Homemaker	108 (56%)	61 (55%)	47 (58%)	
Farmer	34 (18%)	18 (16%)	16 (20%)	0.777
Job holder	32 (16%)	21 (19%)	11 (13%)	
Business	10 (5%)	7 (6%)	3 (4%)	
Unemployed	9 (5%)	5 (4%)	4 (5%)	
Ethnicity				
Aryan	123 (64%)	70 (62%)	53 (65%)	0.676
Mongol	70 (36%)	42 (38%)	28 (35%)	
Education				0.170
Secondary to below	36 (19%)	18 (16%)	18 (22%)	
Higher secondary	125 (65%)	71 (63%)	54 (67%)	
Bachelor and above	32 (16%)	23 (21%)	9 (11%)	
Married	185 (96%)	106 (95%)	79 (98%)	0.321
BMI (kg/m^2^)	27.33 ± 4.06	27.45 ± 3.89	27.21 ± 4.46	0.592
Socioeconomic status				
Lower	10 (5%)	7 (6%)	3 (4%)	
Upper lower	177 (92%)	101 (90%)	76 (94%)	0.662
Middle lower	4 (2%)	2 (2%)	2 (2%)	
Upper middle	2 (1%)	2 (2%)	0	
ASA class				0.002
1	78 (40%)	56 (50%)	22 (27%)	
2	113 (59%)	55 (49%)	58 (72%)	
3	2 (1%)	1 (1%)	1 (1)	
Insurance coverage				0.887
Government	175 (90%)	100 (89%)	75 (93%)	
Private	5 (3%)	3 (3%)	2 (2%)	
Workers' compensation	5 (3%)	4 (3.5%)	1 (1%)	
None	8 (4%)	4 (4.5%)	3 (4%)	
Alcoholic	34 (18%)	15 (13%)	19 (23%)	0.070
Smoker	21 (11%)	9 (8%)	12 (14%)	0.136
Charlson index scores (0–33)	0 (0–1)	0 (0–1)	0 (0–2)	0.003
Diabetes	29 (15%)	9 (8%)	20 (25%)	0.001
Hypertension	53 (27%)	23 (21%)	30 (37%)	0.011
Preoperative analgesic used				<0.001
Paracetamol	3 (2%)	1 (1%)	2 (2%)	
NSAIDS	44 (23%)	10 (9%)	34 (42%)	
Gabapentenoids	4 (2%)	0	4 (5%)	
None	142 (73%)	101 (90%)	41 (51%)	
PROMIS pain intensity scores (3–15)	5 (3–8)	3 (3–3)	8 (7–9)	<0.001
PROMIS pain interference scores (6–30)	16 (14–18)	16 (14–18)	16 (14–18)	0.773
PROMIS pain behavior scores (7–42)	20 (16–24)	18 (16–22)	24 (18–26)	<0.001
PROMIS depression scores (8–40)	16 (14–22)	16 (14–20)	20 (14–18)	<0.001
PROMIS sleep disturbance scores (8–40)	16 (14–22)	16 (14–20)	20 (14–22)	<0.001
APAIS score (6–30)	8 (6–14)	6.5 (6–9)	13 (6–16)	<0.001
Neuroticism (0–12)	1 (0–3)	1 (0–2)	2 (0–3)	<0.001
PCS scores (0–52)	4 (0–8)	2 (0–5)	7 (3–8)	<0.001
Pain expectation (in NPRS)				0.001
Mild (0–3)	120 (62%)	82 (73%)	38 (47%)	
Moderate (4–6)	63 (33%)	27 (24%)	36 (44%)	
Severe (7–10)	10 (5%)	3 (3%)	7 (9%)	
Pain sensitivity (in NPRS)				0.003
Mild (0–3)	178 (92%)	109 (97%)	69 (85%)	
Moderate (4–6)	12 (6%)	2 (2%)	10 (12%)	
Severe (7–10)	3 (2%)	1 (1%)	2 (3%)	
IV fentanyl supplement needed	79 (41%)	37 (33%)	42 (52%)	0.009
IV dexamethasone	188 (97%)	109 (97%)	79 (98%)	1
IV lidocaine	6 (3%)	4 (4%)	2 (2%)	1
IV esmolol	19 (10%)	12 (11%)	7 (9%)	0.633
Parkland grading*				0.020
1	44 (23%)	30 (27%)	14 (17%)	
2	101 (52%)	63 (56%)	38 (47%)	
3	33 (17%)	12 (11%)	21 (26%)	
4	15 (8%)	7 (6%)	8 (10%)	
Incision extension for GB retrieval	28 (15%)	13 (12%)	15 (19%)	0.178
Abdominal drain placement	25 (13%)	13 (12%)	12 (15%)	0.513
Duration of surgery (min)	40 (30–50)	40 (30–50)	45 (35–55)	0.006
Postoperative pain at rest (NPRS)	11 (6%)	4 (4%)	7 (9%)	0.207
Postoperative pain at movement (NPRS)	96 (50%)	45 (40%)	51 (63%)	0.002
Postoperative shoulder pain	17 (9)	9 (8%)	8 (10%)	0.656
Total oral morphine equivalent (mg) up to 24 h	0 (0–12)	0 (0–6)	6 (0–18)	<0.001

Values are expressed as means ± standard deviation, median (interquartile range), or number (%).

* Parkland grading scale: grade 1 = normal appearing gallbladder (GB), no adhesion present; grade 2 = minor adhesion at neck, otherwise normal GB; grade 3 = presence of any of the following (hyperemia, pericholecystic fluid, adhesions to the body, distended GB); grade 4 = presence of any of the following: adhesions obscuring majority of GB, grade1 to grade 3 with abnormal liver anatomy, intrahepatic GB, or impacted stone.

APAIS, Amsterdam preoperative anxiety and information scale; ASA, American Society of Anesthesiologist; BMI, body mass index; IV, intravenous; NPRS, numerical pain rating scale; PCS, pain catastrophizing scale; PROMIS, patient-reported outcome measurement information system.

**Figure 2.**
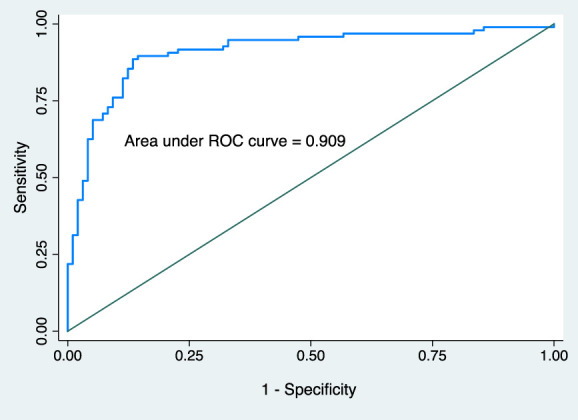
The receiver operating curve (ROC) for the prediction model developed using multivariable logistic regression.

**Figure 3.**
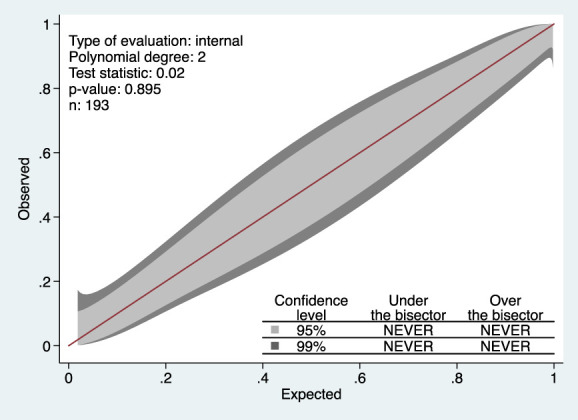
A calibration belt displaying deviations from the 45-degree line of perfect fit, with the 95% confidence level shown as a light gray inner belt and the 99% confidence level shown as a dark gray outer belt.

Table 2 summarizes univariable and multivariable perioperative factors associated with dynamic moderate-to-severe acute postoperative pain. Multivariable analysis revealed that preoperative PROMIS pain intensity score, preoperative PROMIS sleep disturbance score, intraoperative dexamethasone use, intraoperative fentanyl supplementation, incision extension for gallbladder retrieval, and abdominal drain placement were associated with dynamic moderate-to-severe acute pain after LC. No collinearity was observed among the variables. The model's performance, as reflected by the ROC curve for the multivariable logistic regression analysis, was 0.909 (95% CI 0.86–0.95) for predicting moderate-to-severe acute pain after LC (Fig. 2). The mean AUC calculated using 10-fold cross-validation of the final model showed 0.013 points lower accuracy (AUC = 0.896, bootstrap-corrected 95% CI, 0.82–0.92) than the AUC computed using the classical approach from the predicted probabilities of dynamic moderate-to-severe acute pain (AUC = 0.909; Figure S1, Supplementary appendix, http://links.lww.com/PR9/A265). The Hosmer–Lemeshow test results indicated that the goodness-of-fit was satisfactory (χ^2^ = 9.37, 8 degrees of freedom, *P* = 0.153). The calibration belt plot at the 95% and 99% CI encompassed the bisector over the whole range of the predicted probabilities, indicating that there were no ranges of significant miscalibration (Fig. 3). The corresponding *P*-value obtained from the formal statistical test was not significant (test statistic = 0.02; *P* = 0.89), indicating that the model's internal calibration was acceptable.

**Table 2:** Univariable and multivariable analysis of risk factors associated with moderate-to-severe pain after laparoscopic cholecystectomy.

Variable	Univariable		Multivariable	
	OR (95% CI)	*P*	OR (95% CI)	*P*
Age (in y)	0.99 (0.97–1.01)	0.569	0.97 (0.94–1)	0.094
Female	0.64 (0.32–1.28)	0.213	0.56 (0.19–1.63)	0.289
Preexisting chronic pain	2.53 (1.40–4.55)	0.002	0.38 (0.10–1.35)	0.137
Diabetes	4.77 (1.84–12.35)	0.001	2.89 (0.74–11.29)	0.127
Preoperative PROMIS pain intensity	1.21 (1.08–1.36)	0.001	1.28 (1–1.65)	0.043
Preoperative PROMIS sleep disturbance	1.40 (1.27–1.54)	<0.001	1.42 (1.24–1.61)	<0.001
IV dexamethasone received intraoperative	0.23 (0.02–2.18)	0.205	0.05 (0.004–0.74)	0.029
IV fentanyl supplemented intraoperative	7.64 (3.95–14.79)	<0.001	3.68 (1.48–9.12)	0.005
Incision extension for GB retrieval	7.75 (2.57–23.33)	<0.001	7.27 (1.58–33.39)	0.011
Abdominal drain placement	14.96 (3.41–65.53)	<0.001	6.09 (1.08–34.34)	0.041

CI, confidence interval; GB, gallbladder; IV, intravenous; PROMIS, patient-reported outcome measurement information system.

In our post hoc analysis, we further divided patients without preexisting chronic pain into 2 groups: those with subacute pain (duration <3 months) and those without any pain. Among the 112 patients without preexisting chronic pain, 22 patients (11.40%) had subacute pain, while 90 patients (80.36%) had no pain preoperatively. We found that among the 22 patients in the subacute pain group, 12 (54.55%) reported moderate-to-severe acute pain postoperatively. However, in the univariable analysis, no significant association was observed between preexisting subacute pain and postoperative acute moderate-to-severe pain (Tables 1 and 2, Supplementary Appendix, http://links.lww.com/PR9/A265).

## 4. Discussion

In this prospective cohort study, we observed that after adjustment for confounders, there was no significant association between chronic pain before surgery and the occurrence of dynamic moderate-to-severe acute pain after LC. However, in the multivariable analysis, several other factors were identified as independent predictors of dynamic moderate-to-severe acute pain after LC. These factors included preoperative PROMIS pain intensity score, preoperative PROMIS sleep disturbance score, intraoperative dexamethasone use, intraoperative fentanyl supplementation, incision extension for gallbladder retrieval, and abdominal drain placement.

Preoperative pain is associated with both short- and long-term postoperative pain-related outcomes. Studies have consistently shown that preexisting chronic pain is a significant risk factor for chronic postsurgical pain.^2,14,26^ However, the presence of chronic pain as a predictor of acute postoperative pain has yielded conflicting results. A multicenter database analysis reported that preoperative chronic pain at the site of surgery had a higher risk for postoperative acute pain after surgery.^28^ By contrast, no significant relationship was observed between preoperative chronic pain and the intensity of acute postoperative pain.^18,23^ In fact, a meta-analysis aimed at identifying preoperative predictors of poor postoperative pain control in adults undergoing inpatient surgery did not find preexisting chronic pain as a significant predictor.^41^ Interestingly, we observed that the magnitude of preoperative pain was linked with postoperative moderate-to-severe acute pain. Preexisting severity of pain has consistently been shown to predict acute postoperative pain across several studies.^11,35,37^ Moreover, studies have shown a linear relationship between preoperative chronic pain intensity and severe acute postoperative pain.^9,13^ This reflects that the degree of preexisting chronicity is more impactful than the mere presence of chronic pain. Nevertheless, further research is needed to understand the complex relationship between preoperative chronic pain and postoperative acute pain intensity.

In our study, PROMIS sleep disturbance scores emerged as an independent predictor of moderate-to-severe acute pain after LC. Several studies have demonstrated that preoperative sleep deprivation worsens postoperative pain.^18,39,42^ Apart from insomnia, even a brief period of sleep disruption before surgery is associated with increased postoperative pain.^40,42^ Based on experimental and clinical studies, several pathways are affected by poor sleep quality, such as alteration of brainstem opioidergic signaling and elevated inflammatory/stress response due to activation of the hypothalamic–pituitary–adrenal axis, thus exacerbating postsurgical nociception.^36^ Nevertheless, the mechanisms by which sleep deprivation increases postsurgical pain are yet to be elucidated. Despite both sleep and pain being reciprocally related, we observed a weak correlation between preoperative pain intensity and sleep disturbance scores. Further analysis did not reveal any collinearity or interaction between the 2 variables (supplementary appendix, http://links.lww.com/PR9/A265). Therefore, future studies should explore the longitudinal relationship between perioperative sleep disorders and pain outcomes.

We found that patients requiring fentanyl supplementation intraoperatively had an increased incidence of moderate–severe acute pain after surgery. Evidence shows that patients treated with short-acting perioperative opioids are more likely to develop opioid-induced hyperalgesia (OIH), ie, a paradoxical increased sensitivity to pain.^6^ Although OIH may be a possible explanation for intense pain postoperatively in our patients, we did not assess OIH. Besides OIH, escalating the doses of intraoperative opioids is associated with several other adverse outcomes.^7,15^ Therefore, it is suggested to use opioid-sparing agents and minimize the use of opioids perioperatively. Contradictory to our findings, a recent retrospective database study found that increased intraoperative fentanyl was associated with lower maximum pain scores in the PACU, whereas the adjusted odds ratio for 24-hour postoperative pain scores was 1.03 (1.01–1.06).^27^ The authors did not explain the reasons behind the discrepancy between the maximum pain scores at PACU and 24-hour postoperatively with the addition of intraoperative fentanyl. Whether the increased use or reduction of intraoperative opioids heightens postoperative acute pain is a matter of debate; as a result, larger randomized clinical trials are needed to establish causality.

Despite the implementation of multimodal analgesia, nearly half of the patients in our study reported moderate-to-severe acute pain after LC, consistent with the findings of other LC studies. The pain experienced after LC is multifactorial, and therefore, if we can identify patients who are more likely to experience higher levels of postoperative pain, we can implement a more aggressive pain management strategy during the perioperative period. Such an approach would not only enhance the quality of recovery but also help prevent unfavorable outcomes. A notable strength of our study was its comprehensive analysis, which encompassed demographic, psychobehavioral, preoperative, and intraoperative clinical variables. Unlike prior research concentrating on preoperative factors, our approach allowed for an exploration of multiple contributors to moderate-to-severe acute post-LC pain, demonstrating an outstanding prediction ability.

There are several limitations to this study. First, our primary endpoint was limited to 24 hours after surgery. Second, we did not assess an important outcome measure, ie, quality of recovery, as it had not been validated in the Nepali language at the time we initiated our study. Third, our study was confined to LC; therefore, results from our study cannot be extrapolated to other laparoscopic procedures. Finally, although we performed internal validation of our predictive model, it needs to be externally validated.

In conclusion, we did not observe a significant association between preexisting chronic pain and moderate-to-severe acute pain after LC. However, other identified risk factors included higher preoperative pain intensity, sleep disturbances, intraoperative fentanyl requirements, incision extension for gallbladder retrieval, and abdominal drain placement, while dexamethasone use was associated with reduced acute postoperative pain. The predictive model showed excellent discrimination and calibration, suggesting it could help identify patients at higher risk of developing intense acute postoperative pain. Further studies in different clinical settings are required to externally validate our model.

## Disclosures

The authors have no conflict of interest to declare.

## Supplemental digital content

Supplemental digital content associated with this article can be found online at http://links.lww.com/PR9/A265.

## Supplementary Material

SUPPLEMENTARY MATERIAL
