# Supplementary material for: Preexisting chronic pain is not associated with moderate-to-severe acute pain after laparoscopic cholecystectomy: a prospective cohort study
Source: Pain Rep. 2024 Nov 13;9(6):e1214. doi: 10.1097/PR9.0000000000001214 (PMC11563003; doi:10.1097/PR9.0000000000001214)
Supplement: SUPPLEMENTARY MATERIAL [file painreports-9-e1214-s001.pdf]

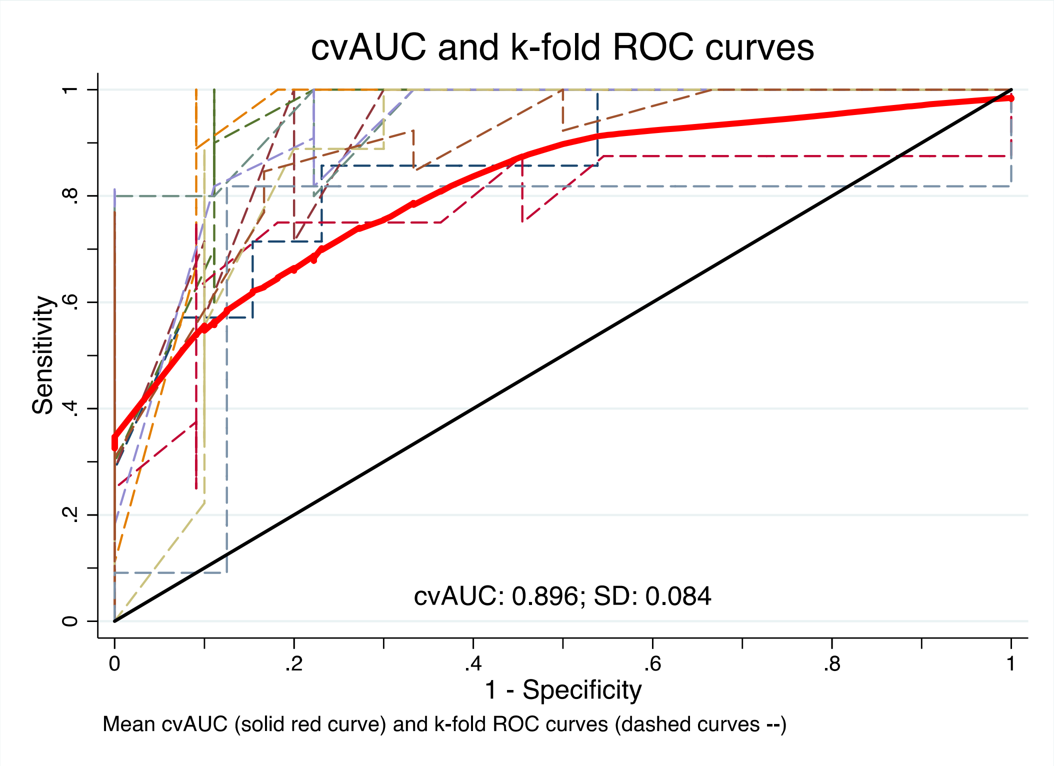

Figure 1. Receiver operator curve (ROC) for logistic regression showing mean cross-validated area under the curve (AUC) resulting after 10-fold-cross validation.

Interaction between preoperative pain intensity and PROMIS sleep disturbances in the model

xi:logistic dynamic_modsevpain age sex diabetes chronic_pain intensity_promis sleep_promis c.intensity_promis#c.sleep_promis fentanyl_intra dexona incision_extension abd_drains

```
Logistic regression                              Number of obs     =        193
                                                 LR chi2(11)       =     122.02
                                                 Prob > chi2       =     0.0000
Log likelihood = -72.766064                      Pseudo R2         =     0.4561

-----------------------------------------------------------------------------------------------------
                 dynamic_modsevpain | Odds Ratio   Std. Err.      z    P>|z|     [95% Conf. Interval]
------------------------------------+----------------------------------------------------------------
                                age |   .9732678   .0158928    -1.66   0.097     .9426116    1.004921
                                sex |   .5605764     .30753    -1.06   0.291     .1912797     1.64286
                           diabetes |   2.905094   2.007754     1.54   0.123     .7496907    11.25741
                       chronic_pain |   .3923397   .2555707    -1.44   0.151     .1094431    1.406489
                   intensity_promis |   1.440056   .5741933     0.91   0.360     .6591432    3.146145
                       sleep_promis |   1.472272   .2079993     2.74   0.006     1.116175    1.941977
                                    |
c.intensity_promis#c.sleep_promis |   .9932768   .0229342    -0.29   0.770     .9493285     1.03926
                                    |
                     fentanyl_intra |   3.717726    1.72651     2.83   0.005     1.496166    9.237935
                             dexona |   .0575087    .074661    -2.20   0.028      .004515    .7325038
                 incision_extension |   7.320192   5.735143     2.54   0.011     1.576277     33.9948
                         abd_drains |    6.05705   5.365655     2.03   0.042     1.067141    34.37958
                              _cons |   .0135338   .0359209    -1.62   0.105     .0000745    2.458339
-----------------------------------------------------------------------------------------------------
Note: _cons estimates baseline odds.
```

Table 1. Patients with acute moderate-severe pain categorised based on preoperative duration of pain.

Preoperative pain	Moderate-severe pain reported
No pain (90 patients)	33 (37%)
Sub-acute pain (< 3 months duration, 22 patients)	12 (55%)
Chronic pain (> 3 months duration, 81 patients)	51 (63%)

Table 2. Univariable analysis of preexisting pain associated with moderate to severe pain after laparoscopic cholecystectomy.

**Variable**	**Univariable**	
	**OR (95% CI)**	**p- value**
Pre-existing pain		
	Ref. No pain preoperatively	
Sub-acute pain (< 3 months duration)	2.07 (0.80-5.31)	0.130
Chronic pain (> 3 months)	2.93 (1.57-5.47)	0.002
